# Out of Africa: the mite community (Arachnida: Acariformes) of the common waxbill, *Estrilda astrild* (Linnaeus, 1758) (Passeriformes: Estrildidae) in Brazil

**DOI:** 10.1186/s13071-017-2230-5

**Published:** 2017-06-21

**Authors:** Fabio Akashi Hernandes, Barry M. OConnor

**Affiliations:** 1 Departamento de Zoologia, Universidade Estadual Paulista, Av. 24-A, 1515, Rio Claro, SP 13506-900 Brazil; 2 Department of Ecology and Evolutionary Biology, Museum of Zoology, University of Michigan, Ann Arbor, MI 48109-1079 USA

**Keywords:** Acari, Feather mites, Systematics, Biogeography, Neotropics, Biodiversity

## Abstract

**Background:**

The common waxbill, *Estrilda astrild* (L., 1758) (Passeriformes: Estrildidae) is a small passerine bird native to Sub-Saharan Africa that has been introduced into several regions of the world.

**Results:**

In the present paper, eight mite species (Acariformes) are reported from this host from Brazil, including three species new to science: *Montesauria caravela* n. sp., *M. conquistador* n. sp. (Proctophyllodidae), *Trouessartia transatlantica* n. sp., *T. minuscula* Gaud & Mouchet, 1958, *T. estrildae* Gaud & Mouchet, 1958 (Trouessartiidae), *Onychalges pachyspathus* Gaud, 1968 (Pyroglyphidae), *Paddacoptes paddae* (Fain, 1964) (Dermationidae) and *Neocheyletiella megaphallos* (Lawrence, 1959) (Cheyletidae). Comparative material from Africa was also studied.

**Conclusions:**

These mites represent at least three morpho-ecological groups regarding their microhabitats occupied on the bird: (i) vane mites (*Montesauria* and *Trouessartia* on the large wing and tail feathers); (ii) down mites (*Onychalges*); and (iii) skin mites (*Paddacoptes* and *Neocheyletiella*). On one bird individual we found representatives of all eight mite species. Although the common waxbill was introduced to the Neotropical region almost two centuries ago, we demonstrate that it still retains its Old World acarofauna and has not yet acquired any representatives of typical Neotropical mite taxa.

## Background

The common waxbill, *Estrilda astrild* (L., 1758) (Passeriformes: Estrildidae) is a small passerine bird native to sub-Saharan Africa that has been introduced into several other regions of the world [1, 2]. Along with the helmeted guineafowl, *Numida meleagris* (Linnaeus, 1858) (Galliformes: Numididae), it is among the very few African birds that have successfully adapted to the Neotropics [1, 3]. It is the only member of the large family Estrildidae (*c.*140 species and subspecies) that occurs in Brazil, the remaining species being restricted to Africa and Australasia [1]. Until now, only a single feather mite species, *Onychalges pachyspathus* Gaud, 1968, has been recorded from this host [4]. In this paper, we report eight mite species (Acariformes) from *E. astrild* from Brazil, including three species new to science.

## Methods

The material from Brazil was collected from two freshly dead specimens of *Estrilda astrild* found in and near the campus of the Universidade Estadual Paulista, Rio Claro, São Paulo state. Comparative material from Africa was recently collected with mistnets by teams from the Field Museum of Natural History, Chicago, USA. Additional specimens, collected from museum skins by the late Dr. W.T. Atyeo, are now housed in the University of Michigan Museum of Zoology. In the Brazilian laboratory, one of us (FAH) examined the host bodies under a stereomicroscope and removed mites from them using a needle. The mites were cleared in 30% lactic acid for 24 h at 50 °C, and mounted in Hoyer’s medium according to the standard technique for small acariform mites [5]. After 5 days at 50 °C, the slides were sealed with varnish. Recently collected African specimens were skeletonized in the field, the feathers and skins placed in individual plastic bags with 95% ethanol and transferred to the laboratory of one of us (BMOC). Mite specimens were collected and processed as above. Drawings and measurements of mites were made with a Leica DM3000 microscope equipped with differential interference contrast (DIC) optics and a camera lucida. Pencil sketches were scanned at 300 dpi grayscale, and line drawings were created with Adobe Illustrator CS6 and a Wacom Bamboo Create tablet. The chaetotaxy of the idiosoma and legs follows Griffiths et al. [6] and Atyeo & Gaud [7], respectively, with corrections of coxal setae proposed by Norton [8].

Specimens are deposited in the following collections: Collection of Acari of Department of Zoology of the Universidade Estadual Paulista, Rio Claro, São Paulo State, Brazil (DZUnesp-RC); Zoological Institute, Russian Academy of Sciences, Saint Petersburg, Russia (ZISP); Field Museum of Natural History, Chicago, Illinois, USA (FMNH); Museum of Zoology, the University of Michigan, Ann Arbor, USA (UMMZ); U.S. National Museum of Natural History, Smithsonian Institution; mite collection housed with the U.S. Department of Agriculture, Systematic Entomology Laboratory, Beltsville, Maryland, USA (USNM).

## Results

Eight mite species (Acariformes) belonging to five families were found on the common waxbill in Brazil: *Montesauria caravela* n. sp., *Montesauria conquistador* n. sp. (Proctophyllodidae), *Trouessartia transatlantica* n. sp., *T. minuscula* Gaud & Mouchet, 1958, *T*. *estrildae* Gaud & Mouchet, 1958 (Trouessartiidae), *Onychalges pachyspathus* Gaud, 1968 (Pyroglyphidae), *Paddacoptes paddae* (Fain, 1964) (Dermationidae) and *Neocheyletiella megaphallos* (Lawrence, 1959) (Cheyletidae). One specimen of the common waxbill hosted all eight mite species. All except *P. paddae* and *N. megaphallos* were also recovered from the recently collected African birds. African birds also harbored an undescribed species of *Xolalgoides* (Xolalgidae). The mites are systematically arranged below.

**Superorder Acariformes Zachvatkin, 1952**

**Order Sarcoptiformes Reuter, 1909**

**Infraorder Astigmata Canestrini, 1891**

**Superfamily Analgoidea Trouessart & Mégnin, 1884**

**Family Proctophyllodidae Mégnin & Trouessart, 1884**

**Subfamily Pterodectinae Park & Atyeo, 1971**

**Genus** ***Montesauria*** **Oudemans, 1905**

### Remarks

The type-species of the genus *Montesauria* is *Proctophyllodes* (*Pterodectes*) *cylindricus* Robin, 1877, by original designation, described from the Eurasian Magpie, *Pica pica* (Linnaeus, 1758) (Passeriformes: Corvidae). This is the most species-rich genus of the subfamily Pterodectinae, currently including 60 described species distributed almost exclusively in the Old World [9–15]. The only representative from the New World is *M. reticulifera* (Trouessart & Neumann, 1888), described from *Eremophila alpestris* (Linnaeus, 1758) (Alaudidae) of North America [10]. With the exception of two species associated with non-passerine hosts, *M. centropa* (Gaud & Mouchet, 1957) from African barbets (Piciformes: Lybiidae) [11] and *M. ralliculae* (Atyeo & Gaud, 1977) from New Guinea rails (Gruiformes: Rallidae) [9, 12], all remaining species are associates of passerines (Passeriformes).

Mironov [13] divided the genus into nine species groups and provided a diagnosis for each group based mainly on the following morphological characters: presence/absence of setae *f2*, sclerotization of coxal fields I-II, and the position of dorsal setae *c2* either on the hysteronotal shields or on the surrounding integument. Mironov et al. [14] further proposed one additional group, *macronoi*, to accomodate *M. macronoi* Mironov, Literák, Hung & Čapek, 2012 and *M. pellornei* Mironov, Literák, Hung & Čapek, 2012, and Mironov & Tolstenkov [15] added one more species to this latter group.

***Montesauria caravela*** **n. sp.**

***Type-host*** **:** *Estrilda astrild* (Linnaeus, 1758) (Passeriformes: Estrildidae).

***Type-locality*** **:** Campus of UNESP (22°24′S, 47°33′W), Rio Claro, São Paulo State, Brazil.

***Other localities*** **:** D.R. Congo, South Africa, Mozambique.

***Type-material*** **:** Holotype male, 13 male and 14 female paratypes ex *Estrilda astrild* (Linnaeus, 1758) (Passeriformes: Estrildidae), BRAZIL: São Paulo State, Campus of UNESP, Rio Claro, 22°24′S, 47°33′W, 6.v.2015, M.H. Gabriel coll.; 1 female, same host species, BRAZIL: Rio Claro, São Paulo State, 13 January 2016, C.O.A. Gussoni coll.; 2 males and 5 females ex *E. a. adesma* Reichenow, 1916, D.R. CONGO: South Kivu, Centre de Recherche en Sciences Naturelles, Lwiro, 1702 m, 02°14′S, 28°48′E, 13.vi.2001, J.S. Hunt (JSH 031), FMNH 429841, BMOC 02–0625-011; 1 male and 1 female ex *E. a. astrild* (Linnaeus, 1758), SOUTH AFRICA: Western Cape Province, Mossel Bay, 34°10′59″S, 22°07′42″E, 8.xii.1953, F. Zumpt (NU 3595); 1 male ex *E. a. cavendishi* Sharpe, 1900, MOZAMBIQUE: Sofala Province, Búzi, NW Beira, 19°52′53″S, 34°36′03″E, 8.xi.1961(NU 4190).

***Type-depositories*** **:** Holotype (# 3774), 7 male and 9 female paratypes at DZUnesp-RC (# 3775–3790); 2 male and 2 female paratypes at each UMMZ, USNM and ZISP. African specimens in FMNH and UMMZ.

***ZooBank registration*** **:** To comply with the regulations set out in article 8.5 of the amended 2012 version of the *International Code of Zoological Nomenclature* (ICZN) [16], details of the new species have been submitted to ZooBank. The Life Science Identifier (LSID) of the article is urn:lsid:zoobank.org:pub:0CE77EA6-7DAC-41D4-91CF-EEF74046677F. The LSID for the new name *Montesauria caravela* n. sp. is urn:lsid:zoobank.org:act:C9652592-CDAA-4D5C-A699-EA5A77A0968C.

***Etymology*** **:** From Portuguese *caravela* (= caravel), the sailing ships used by fifteenth century Portuguese explorers in the Age of Discovery. The name is a noun in apposition.

### Description

**Fig. 1:**
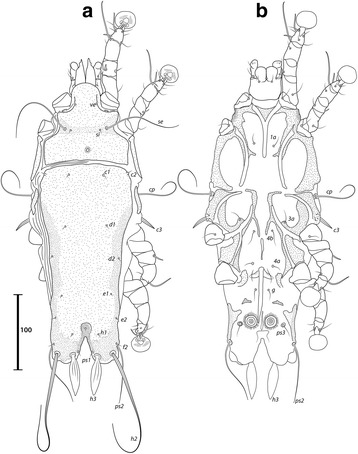
*Montesauria caravela* n. sp., male. **a** Dorsal view. **b** Ventral view

**Fig. 2:**
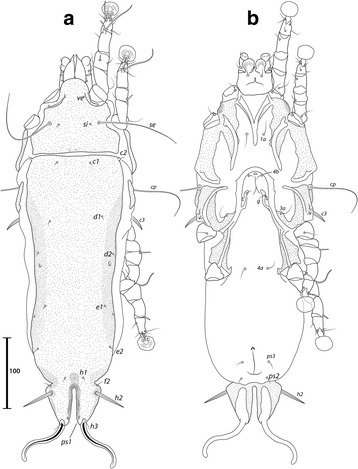
*Montesauria caravela* n. sp., female. **a** Dorsal view. **b** Ventral view

**Fig. 3:**
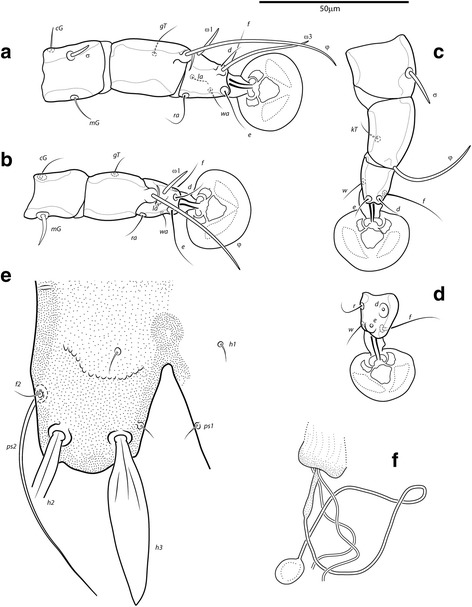
*Montesauria caravela* n. sp., male (**a**-**e**), female (**f**). **a**-**c** Genu, tibia and tarsus of legs I-III. **d** Tarsus IV. **e** Opisthosomal lobe, dorsal view. **f** Spermatheca

***Male.*** [Holotype, range for 9 paratypes in parentheses; Figs. 1, 2 and 3.] Idiosoma length from anterior end of prodorsal shield to posterior margins of lobes 392 (376–394), greatest width of idiosoma at level of humeral shields 150 (146–157). Prodorsal shield entire, lateral margins slightly concave at level of scapular setae, posterior margin straight, length along midline 111 (102–111), greatest width 118 (111–127), surface smooth, with a circular medial darker ornamentation near posterior margin (Fig. 1a); bases of scapular setae *se* separated by 60 (60–65). Setae *ve* present, rudimentary. Scapular shields narrow. Humeral shields present, narrow. Setae *c2* situated at anterolateral margins of hysteronotal shield, setae *cp* on striated tegument. Subhumeral setae *c3* lanceolate, 30 (29–32) × 6 (4–6). Hysteronotal shield: greatest length 274 (272–278), width at anterior margin 122 (125–133), anterior margin slightly convex, surface without lacunae. Distance between prodorsal and hysteronotal shields 7 (3–10). Posterior margins of opisthosomal lobes round. Terminal cleft shaped as an inverted V, 37 (38–41) long. Supranal concavity distinct. Setae *f2* anterior to bases of setae *ps2*. Setae *h1* situated at midlevel of supranal concavity. Setae *h3* leaf-like, 55 (55–63) long; setae *ps2* 77 (75–86) long; setae *ps1* filiform, about 8 long, situated slightly anterior to bases of setae *h3*. Distances between dorsal setae: *c1*:*d2* 118 (105–120), *d2*:*h1* 111 (106–117), *h2*:*h2* 80 (73–80), *h3*:*h3* 35 (30–36).

Coxal apodemes I (epimerites of Gaud & Atyeo [17]) fused into a Y, with sclerotization present between anterior arms (Fig. 1b). Coxal fields I-III with narrow areas of sclerotization. Rudimentary sclerites rEpIIa absent. Coxal fields I-IV open. Coxal apodemes IVa present, at level of genital arch. Genital arch 31 (26–33) in width; aedeagus 79 (73–82) long from anterior bend to tip, extending to midlevel of adanal suckers. Genital papillae not connected at bases. Posterior coxal apodemes with narrow bands of sclerotization. Genital shield as a thin sclerite medially between setae *4b* and *4a*; adanal shields as two small sclerites anterior to adanal suckers. Adanal suckers 16 (15–19) in diameter, distance between centers of discs 28 (21–26), corolla smooth, surrounding membrane with radial striae. Opisthoventral shields occupying lateral areas of opisthosoma; setae *ps3* inserted on membranous integument. Setae *4b* situated posterior to level of setae *3a*. Distance between ventral setae: *1a*:*4b* 122 (112–130), *4b*:*4a* 45 (43–53), *4a*:*g* 34 (30–34), *g*:*ps3* 51 (48–51), *ps3*:*ps3* 59 (60–64).

Femora I, II without crests, other segments of legs I-IV without processes. Ambulacra rounded. Solenidion σ1 of genu I 9 (8–9) long, situated at midlevel of segment; solenidion σ of genu III inserted in distal half of segment. Genual setae *cG*I, II and *mG*I filiform, *mG*II spiculiform. Seta *d* of tarsus II slightly shorter than corresponding seta *f* (Fig. 3b); seta *d* of tarsus III about half of the length of corresponding seta *f* (Fig. 3c). Solenidion φ of tibia IV extending to apex of ambulacral disc. Tarsus IV 21 (20–24) long, without claw-like apical process; setae *d* and *e* button-like, seta *d* situated at basal half of segment (Fig. 3d).

***Female*** **.** [Range for 8 paratypes.] Idiosoma length from anterior end of prodorsal shield to posterior margin of lobes (excluding terminal appendages) 498–513, greatest width of idiosoma at level of humeral shields 153–176. Prodorsal shield: shape as in male, length × width 106–111 × 122–129; surface smooth, with a pair of small, circular spots of ornamentation near posterior margin; bases of setae *se* separated by 115–130 (Fig. 2a). Setae *ve* present, rudimentary. Scapular shields narrowly developed dorsally. Humeral shields narrow. Setae *c2* inserted at anterolateral margins of hysteronotal shield, setae *cp* located ventrally on integument adjacent to humeral shield. Setae *c3* lanceolate, 27–31 × 4–5. Anterior and lobar parts of hysteronotal shield fused (Fig. 2a). Distance between prodorsal and anterior hysteronotal shields 0–3. Anterior margin of hysteronotal shield straight, length from anterior margin to lobar apices (excluding terminal appendages) 386–396, greatest width at anterior part 133–142, surface without lacunae. Lobar region greatest width 78–85. Terminal cleft as a narrow, inverted U, 55–60 long. Supranal concavity distinct, at the same level of setae *h1*; setae *h1* and *f2* arranged in a low trapezium. Setae *h2* lanceolate with sharp apex, 45–51 × 4–5. Setae *h3* 12–15 long. Setae *ps1* situated near inner margins of opisthosomal lobes, closer to *h3* than to *h2*. Distances between dorsal setae: *c1*:*d2* 122–131, *d2*:*h1* 170–182, *h2*:*h2* 66–70, *h3*:*h3* 29–33.

Coxal apodemes I fused into a Y, sternum about half of the total length of these apodemes, anterior arms with small bands of sclerotization between them (Fig. 2b). Coxal fields I-IV open, with narrow areas of sclerotization. Coxal apodemes IVa small. Translobar apodemes of opisthosomal lobes present, narrow, fused to each other anterior to terminal cleft. Epigynum horseshoe shaped, greatest width 48–58; apodemes of ovipore connected with coxal apodemes IIIa. Primary spermaduct with thickened part near the head of spermatheca; secondary spermaducts 48–58 long (Fig. 3f). Pseudanal setae filiform, setae *ps2* situated posterior to the level of the anal opening; distance between pseudanal setae: *ps2*:*ps2* 30–39, *ps3*:*ps3* 25–37, *ps2*:*ps3* 24–32.

Femur II with ventral crest, other segments of legs I, II without processes. Solenidion σ1 of genu I short, 7–10 long, situated at midlevel of segment. Solenidion σ of genu III inserted basally. Genual setae *cG*I, II, *mG*I, II as in male. Setae *f* of tarsi I, II slightly longer than corresponding seta *d*, setae *f* of tarsi III, IV 2–3 times longer than corresponding setae *d*. Genu IV dorsally inflated, without crest.

### Differential diagnosis

*Montesauria caravela* n. sp. belongs to the *emberizae* group, a group characterized by having dorsal setae *c2* inserted on anterolateral margins of hysteronotal shield, setae *f2* present, and coxal fields I-II without large sclerotized areas [13]. Five species are currently included in this group: *M. emberizae* Mironov & Kopij, 1997, *M. faini* Mironov, 2008, *M. stephanocaulus* (Gaud, 1953), *M. tetralobula* Mironov & Kopij, 1996, and *M. zosteropis* Mironov, Literák, Čapek & Koubek, 2010. The new species most closely resembles *M. zosteropis* in having lanceolate setae *h3* in males, but is distinguished from this and from all previously known species of the *emberizae* group by the following features: in both sexes, humeral shields present dorsally; in males, a pair of adanal shields present anterior to adanal suckers, seta *h3* is much wider than in male *M. zosteropis*; in females, anterior hysteronotal and lobar shields fused (these shields separated from each other in all previous species of the *emberizae* group).

***Montesauria conquistador*** **n. sp.**

***Type-host*** **:** *Estrilda astrild* (Linnaeus, 1758) (Passeriformes: Estrildidae).

***Type-locality*** **:** Campus of UNESP (22°24′S, 47°33′W), Rio Claro, São Paulo State, Brazil.

***Other localities*** **:** Gabon, Burundi, Ethiopia, South Sudan, South Africa, Mozambique.

***Type-material*** **:** Holotype male, 9 male and 13 female paratypes ex *Estrilda astrild* (Linnaeus, 1758) (Passeriformes: Estrildidae), BRAZIL: São Paulo State, Campus of UNESP, Rio Claro, 22°24′S, 47°33′W, 06.v.2015, M.H. Gabriel coll.; 8 males and 8 females, same host species, BRAZIL: Rio Claro, São Paulo State, 13 January 2016, C.O.A. Gussoni coll.; 2 males, 2 females and 2 nymphs ex *E. a rubriventris* (Vieillot, 1817), GABON: Ngounié, Agouma, River Nkomi, 01°32′29″S, 10°11′14″E, 7.xii.1917, C.R. Aschemeier, USNM 255788 (UGA 3138); 5 males and 4 females ex *E. a. rubriventris*, GABON: Ngounié, Agouma, River Nkomi, 01°32′29″S, 10°11′14″E, 24.xi.1918, C.R. Aschemeier, USNM 255787 (UGA 3139); 3 females and 1 nymph ex *E. a. adesma* Reichenow, 1916, BURUNDI: Makamba, Nyanza-Lac, 04°20′08″S, 29°35′43″E, 16.iii.1920, H.C. Raven, USNM 275771 (UGA 3141); 3 males, 2 females, 3 nymphs ex *E. a. peasei* Shelley, 1903, ETHIOPIA: Oromiya, Aleta, 09°46′N, 38°45′E, 11.iii.1912, E.A. Mearns, USNM 247464 (UGA 3142); 2 males, 1 nymph ex *E. a. macmillani* Ogilvie-Grant, 1907, SOUTH SUDAN: Jubek State, Gondokoro, 04°54′09″N, 31°39′46″E, 23.ii.1910, E.A. Mearns, USNM 217328 (UGA 3144); 2 males, 1 female ex *E. a. macmillani*, same data as previous, USNM 217329 (UGA 3145); 2 males ex *E. a. macmillani*, same data as previous, 21.ii.1910, USNM 217327 (UGA 3146); 1 male and 1 female ex *E. a. astrild* (Linnaeus, 1758), SOUTH AFRICA: Western Cape Prov., Mossel Bay. 34°10′59″S, 22°07′42″E, 8.xii.1953, F. Zumpt (NU 3595); 3 females ex *E. a. astrild*, SOUTH AFRICA: Western Cape Prov., Cape Town, Rondevlei Bird Sanctuary, 34°03′38″S, 18°29′46″E, 24.ix.1955, E. Middlemiss (NU 3805); 2 females ex *E. a. cavendishi* Sharpe, 1900, MOZAMBIQUE: Sofala Prov., Búzi, NW Beira, 19°52′53″S, 34°36′03″E, 8.xi.1961, (NU 4190); 10 males, 11 females ex *E. a. cavendishi*, MALAWI: Northern Region, Rumphi dist., Khuta maji, Vwaza Marsh, Vwaza Wildlife Reserve, 1170 m, 10°52′S, 33°27′E, 1.x.2009, J.M. Bates, FMNH 489300 (BMOC 10–0503-001); 2 males ex *E. a. cavendishi*, same data as previous, 14.x.2009, J.W. Weckstein, FMNH 489301 (BMOC 10–0503-003).

***Type-depositories*** **:** Holotype (# 3791), 10 male and 15 female paratypes at DZUnesp-RC (# 3792–3816); 2 male and 2 female paratypes at each UMMZ, USNM and ZISP. African specimens in FMNH, UMMZ and USNM.

***ZooBank registration*** **:** To comply with the regulations set out in article 8.5 of the amended 2012 version of the *International Code of Zoological Nomenclature* (ICZN) [16], details of the new species have been submitted to ZooBank. The Life Science Identifier (LSID) of the article is urn:lsid:zoobank.org:pub:0CE77EA6-7DAC-41D4-91CF-EEF74046677F. The LSID for the new name *Montesauria conquistador* n. sp. is urn:lsid:zoobank.org:act:2C0E9CAE-15EC-456E-8E59-0BDAF6BA2E53.

***Etymology*** **:** From Portuguese *conquistador* (= *conqueror*), an allusion to the Portuguese and Spanish “conquistadores” of the 16th–18th centuries who vastly explored the globe and founded new populations, mainly in the New World. The name is a noun in apposition.

**Fig. 4:**
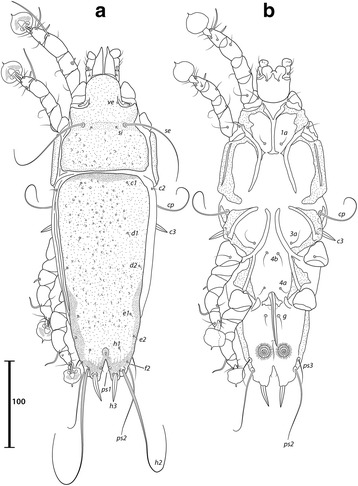
*Montesauria conquistador* n. sp., male. **a** Dorsal view. **b** Ventral view

**Fig. 5:**
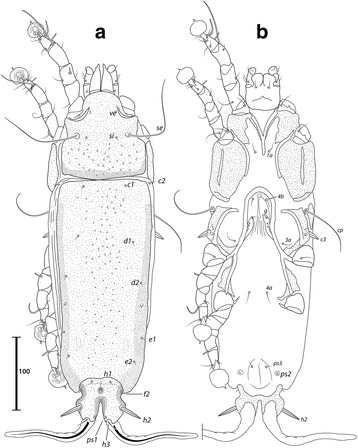
*Montesauria conquistador* n. sp., female. **a** Dorsal view. **b** Ventral view

**Fig. 6:**
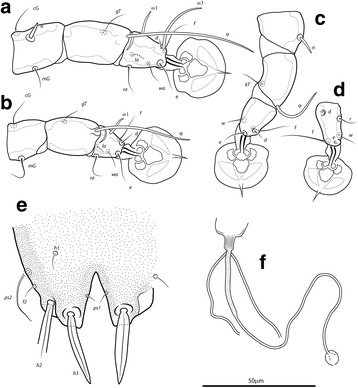
*Montesauria conquistador* n. sp., male (**a**-**e**), female (**f**). **a**-**c** Genu, tibia and tarsus of legs I-III. **d** Tarsus IV. **e** Opisthosomal lobe, dorsal view. **f** Spermatheca

***Male.*** [Holotype, range for 7 paratypes in parentheses; Figs. 4, 5 and 6.] Idiosoma length from anterior end of prodorsal shield to posterior margins of lobes 368 (362–379), greatest width of idiosoma at level of humeral shields 128 (119–138). Prodorsal shield: entire, lateral margins slightly concave at level of scapular setae, posterior margin straight, length along midline 111 (106–110), greatest width 103 (102–109), surface with faint and scarce circular lacunae distributed posterior to level of scapular setae (Fig. 4a); bases of scapular setae *se* separated by 50 (47–52). Setae *ve* present, rudimentary. Scapular shields narrow. Humeral shields present as narrow ventral sclerites adjacent to bases of seta *cp*. Setae *c2* situated on the striated integument, setae *cp* ventrally on striated integument. Subhumeral setae *c3* lanceolate, 22 (23–25) × 6 (5–6). Hysteronotal shield: greatest length 246 (239–262), width at anterior margin 109 (105–117), anterior margin slightly convex, surface with scarce circular lacunae, anterior margin with slightly darker patch of sclerotization. Distance between prodorsal and hysteronotal shields 4 (4–7). Posterior margins of opisthosomal lobes afilate. Terminal cleft shaped as an inverted V, 27 (27–30) long. Supranal concavity distinct. Setae *f2* anterior to bases of setae *ps2*. Setae *h1* situated at the anterior level of supranal concavity. Setae *h3* lanceolate, 35 (34–40) long; setae *ps2* 77 (74–90) long; setae *ps1* filiform, about 4 long, situated slightly anterior to bases of setae *h3*. Distances between dorsal setae: *c1*:*d2* 95 (90–100), *d2*:*h1* 96 (95–101), *h2*:*h2* 44 (37–44), *h3*:*h3* 25 (18–23).

Coxal apodemes I fused into a Y, posterior end of sternum with lateral extensions not connected with coxal apodemes II, sclerotization present between anterior arms, (Fig. 4b). Coxal fields I-III with narrow areas of sclerotization. Rudimentary sclerites rEpIIa absent. Coxal fields I-IV open. Coxal apodemes IVa present, at level of genital arch. Genital arch 20 (21–26) in width; aedeagus 64 (63–69) long from anterior bend to tip, extending to the anterior level of adanal suckers. Genital papillae not connected at bases. Posterior coxal apodemes with narrow bands of sclerotization. Genital and adanal shields absent. Adanal suckers 14 (13–15) in diameter, distance between centers of discs 24 (22–25), corolla indented, surrounding membrane with radial striae. Opisthoventral shields occupying lateral areas of opisthosoma to bases of *ps2*; setae *ps3* inserted on the margins of opisthoventral shield. Setae *4b* situated posterior to level of setae *3a*. Distance between ventral setae: *1a*:*4b* 127 (124–134), *4b*:*4a* 43 (38–45), *4a*:*g* 33 (29–34), *g*:*ps3* 52 (50–58), *ps3*:*ps3* 58 (54–62).

Femora I, II with ventral crests (Fig. 4b), other segments of legs I-IV without processes. Ambulacra with pointed median axis. Solenidion σ1 of genu I 8 (7–8) long, situated at basal half of segment; solenidion σ of genu III inserted at midlevel of segment. Genual setae *cG*I, II and *mG*I, II filiform. Seta *f* of tarsi II about twice the length of corresponding seta *d* (Fig. 6b); seta *f* of tarsi III three times longer than corresponding seta *d* (Fig. 6c). Solenidion φ of tibia IV extending to midlevel of apex of ambulacral disc. Tarsus IV 25 (24–26) long, with small claw-like apical process; setae *d* and *e* button-like, seta *d* situated at basal half of segment (Fig. 6d).

***Female***. [Range for 8 paratypes.] Idiosoma length from anterior end of prodorsal shield to posterior margin of lobes (excluding terminal appendages) 459–497, greatest width of idiosoma at level of humeral shields 148–172. Prodorsal shield: shape as in male, length × width 119–129 × 116–126, surface with scarcely distributed circular lacunae posterior to the level of scapular setae, posterior margin of the shield with darker patch of sclerotization, bases of setae *se* separated by 63–75 (Fig. 5a). Setae *ve* present, rudimentary. Scapular shields narrowly developed dorsally. Humeral shields narrow. Setae *c2* inserted on the striated integument, setae *cp* ventrally on integument adjacent to humeral shield. Setae *c3* lanceolate, 20–24 × 6–7. Anterior and lobar parts of hysteronotal shield not connected (Fig. 5b). Distance between prodorsal and anterior hysteronotal shields 0–6. Anterior margin of hysteronotal shield straigth, length from anterior margin to lobar apices (excluding terminal appendages) 286–312, greatest width at anterior part 130–149, surface with a few lacunae medially. Lobar region greatest width 57–73. Terminal cleft as a narrow, inverted U, 30–36 long. Supranal concavity distinct, at the same level of setae *f2*; setae *h1* and *f2* arranged in a low trapezium. Setae *h2* lanceolate with blunt apex, 34–40 × 5–6. Setae *h3* 14–18 long. Setae *ps1* situated near inner margins of opisthosomal lobes, closer to *h3* than to *h2*. Distances between dorsal setae: *c1*:*d2* 113–134, *d2*:*h1* 146–163, *h2*:*h2* 54–64, *h3*:*h3* 31–36.

Coxal apodemes I fused into a Y, sternum about half of the total length of these apodemes, anterior arms connected with sclerotized parts of coxal fields I (Fig. 5b). Coxal fields I open, coxal fields II covered with sclerotization except for longitudinal fissure, coxal apodemes III, IV with narrow areas of sclerotization. Coxal apodemes IVa small. Translobar apodemes of opisthosomal lobes present, narrow, fused to each other anterior to terminal cleft. Epigynum horseshoe shaped, greatest width 43–50; apodemes of ovipore connected with coxal apodemes IIIa. Primary spermaduct thickened in the proximal third; secondary spermaducts 38–48 long (Fig. 6f). Pseudanal setae *ps3* filiform, setae *ps2* button-like, situated at midlevel of anal opening; distance between pseudanal setae: *ps2*:*ps2* 50–59, *ps3*:*ps3* 20–24, *ps2*:*ps3* 2–10.

Femur II with ventral crest, other segments of legs I, II without processes. Solenidion σ1 of genu I short, 8–9 long, situated at midlevel of segment. Solenidion σ of genu III inserted basally. Genual setae *cG*I, II, *mG*I, II as in male. Seta *f* of tarsi I, II slightly longer than corresponding seta *d*, setae *f* of tarsi III, IV 2–3 times longer than corresponding setae *d*. Genu IV dorsally inflated, without crest.

### Differential diagnosis

*Montesauria conquistador* n. sp. belongs to the *heterocaula* species group, which is so far restricted to the Estrildidae and is characterized by having dorsal setae *c2* off the hysteronotal shield, setae *f2* present, and coxal fields I-II with large sclerotized areas [13]. Seven species are currently recognized in this group: *M. bacillus* (Trouessart, 1885), *M. heterocaula* (Gaud & Mouchet, 1957), *M. lanceolatus* (Sugimoto, 1941), *M. nesocharis* Mironov & Fain, 2003, *M. olygosticta* (Gaud & Mouchet, 1957), *M. stictothyra* (Gaud, 1953), and *M. synosterna* (Gaud & Mouchet, 1957). *Montesauria conquistador* n. sp. most closely resembles *M. heterocaula* in the overall body shape of females (body length about three times body width). It is readily distinguished from *M. heterocaula* in the following features: in females, setae *ps2* button-like (normal, setiform in *M. heterocaula*); in males, sclerotization between arms of coxal apodemes I is absent (present in *M. heterocaula*).

**Family Trouessartiidae Gaud, 1957**

**Genus** ***Trouessartia*** **Canestrini, 1899**

### Remarks

The type-species of *Trouessartia* is *Dermaleichus corvinus* Koch, 1841, designated subsequently by Oudemans [18] as the type-species of *Pterocolus* Haller, 1878 (junior homonym of *Pterocolus* Schoenherr, 1833, Insecta: Curculionidae). With the exception of one species reliably reported from woodpeckers (Piciformes: Picidae) [19], all remaining species are associated with the order Passeriformes worldwide. 117 species are currently known in this genus [19–30].

***Trouessartia transatlantica*** **n. sp.**

***Type-host*** **:** *Estrilda astrild* (Linnaeus, 1758) (Passeriformes: Estrildidae).

***Type-locality*** **:** Campus of UNESP (22°24′S, 47°33′W), Rio Claro, São Paulo State, Brazil.

***Other localities*** **:** Burundi, D.R. Congo, South Sudan, South Africa, Mozambique, Malawi, Mauritius.

***Type-material*** **:** Holotype male, 13 male and 14 female paratypes ex *Estrilda astrild* (Linnaeus, 1758) (Passeriformes: Estrildidae), BRAZIL: São Paulo State, Campus of UNESP, Rio Claro, 22°24′S, 47°33 W′, 06.v.2015, M.H. Gabriel coll.; 1 male and 1 female, same host species, BRAZIL: Rio Claro, São Paulo State, 13 January 2016, C.O.A. Gussoni coll.; 1 male ex *E. a. adesma* Reichenow, 1916, BURUNDI: Makamba, Nyanza-Lac, 04°20′08″S, 29°35′43″E, 16.iii.1920, H.C. Raven, USNM 275771 (UGA 3141); 4 males and 3 females ex *E. a. adesma*, D. R. CONGO: South Kivu, Centre de Recherche en Sciences Naturelles, Lwiro, 1702 m, 02°14′S, 28°48′E, 13.vi.2001, J.S. Hunt (JSH 031), FMNH 429841 (BMOC 02–0625-011); 1 male and 2 females ex *E. a. macmillani* Ogilvie-Grant, 1907, SOUTH SUDAN: Jubek State, Gondokoro, 04°54′09″N, 31°39′46″E, 23.ii.1910, E.A. Mearns, USNM 217328 (UGA 3144); 1 male ex *E. a. macmillani*, same data as previous, USNM 217329 (UGA 3145); 2 males and 1 female ex *E. a. macmillani*, same data as previous, 21.ii.1910, USNM 217327 (UGA 3146); 1 male and 1 female ex *E. a. astrild* (Linnaeus, 1758), SOUTH AFRICA: Western Cape Prov., Mossel Bay. 34°10′59″S, 22°07′42″E, 8.xii.1953, F. Zumpt (NU 3595); 1 female ex *E. a. astrild*, same data as previous (NU 3604); 3 males and 3 females ex *E. a. cavendishi* Sharpe, 1900, MOZAMBIQUE: Sofala Prov., Búzi, NW Beira, 1952′53″S, 34°36′03″E, 8.xi.1961, (NU 4190); 1 female ex *E. a. cavendishi*, MALAWI: Northern Region, Rumphi dist., Khuta maji, Vwaza Marsh, Vwaza Wildlife Reserve, 1170 m, 10°52′S, 33°27′E, 1.x.2009, J.M. Bates, FMNH 489300 (BMOC 10–0503-001); 1 male and 2 females ex *E. a. cavendishi*, same data as previous, 14.x.2009, J.W. Weckstein, FMNH 489301 (BMOC 10–0503-003); 3 males and 2 females ex *E. a. cavendishi*, MAURITIUS: Rodrigues Is., 19°42′S, 63°25′E, 1.x.1964, F.B. Gill, USNM 486951 (UGA 3173); 2 males ex *E. a. cavendishi*, same data as previous (UGA 3173); 4 males and 1 female ex *E. a. cavendishi*, MAURITIUS: Mauritius Is., Savanne Dist., nr. Bel Ombre, 20°30′03″S, 57°24′22″E, 15.ix.1964, F.B. Gill, USNM 487194 (UGA 3175); 3 males and 5 females ex *E. a. cavendishi*, French Overseas Department: Reunion Island, Étang-Salé les Bains, 21°16′05″S, 55°20′03″E, 19.ix.1964, F.B. Gill, USNM 487040 (UGA 3176).

***Type-depositories*** **:** Holotype (# 3817), 8 male and 9 female paratypes (# 3818–3834) at DZUnesp-RC; 2 male and 2 female paratypes at each UMMZ, USNM, ZISP. African specimens in FMNH, UMMZ, USNM.

***ZooBank registration*** **:** To comply with the regulations set out in article 8.5 of the amended 2012 version of the *International Code of Zoological Nomenclature* (ICZN) [16], details of the new species have been submitted to ZooBank. The Life Science Identifier (LSID) of the article is urn:lsid:zoobank.org:pub:0CE77EA6-7DAC-41D4-91CF-EEF74046677F. The LSID for the new name *Trouessartia transatlantica* n. sp. is urn:lsid:zoobank.org:act:DED23D1D-9157-42B6-BA01-ADFADBB93EE2.

***Etymology*** **:** The specific epithet refers to the two continents separated by the Atlantic Ocean - Africa and South America - in which the common waxbill now occurs. The species name is in the form of an adjective.

**Fig. 7:**
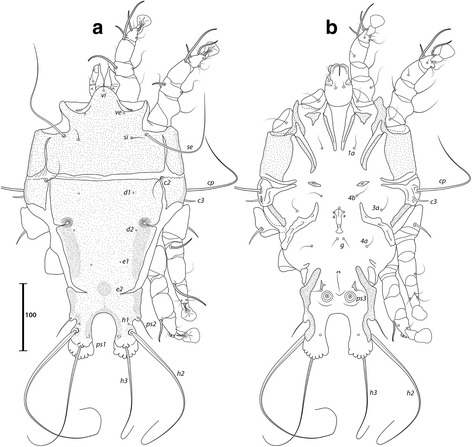
*Trouessartia transatlantica* n. sp., male. **a** Dorsal view. **b** Ventral view

**Fig. 8:**
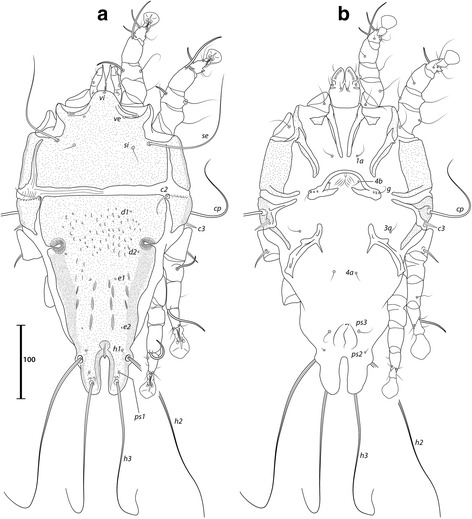
*Trouessartia transatlantica* n. sp., female. **a** Dorsal view. **b** Ventral view

***Male***. [Holotype, range for 4 paratypes in parentheses; Figs. 7 and 8.] Length of idiosoma from anterior end to bases of setae *h3* 409 (401–414), greatest width of idiosoma at level of humeral shields 242 (237–242). Length of hysterosoma from sejugal furrow to bases of setae *h3* 255 (247–258). Prodorsal shield: length along midline 126 (124–129), greatest width of posterior part 173 (168–177), anterior part at level of trochanters II narrowed, lateral margins fused with scapular shields, posterior margin straight, surface smooth (Fig. 7a). Vertical setae *ve* as microseta. Internal scapular setae *si* thin piliform, 27 (22–29) long, separated by 82 (75–82); external scapular setae *se* 158 (133–159) long, separated by 122 (121–125). Humeral shield with setae *c2* thin, 47 (31–50) long. Setae *c3* narrowly lanceolate, acute apically, 19 (19–26) long. Prohysteronotal and lobar shields connected medially. Prohysteronotal shield: length 181 (170–181), width at widest part near the anterior margin 171 (166–176), lateral margins with shallow incisions at level of trochanters III, dorsal hysterosomal apertures (DHA) present, surface smooth. Dorsal setae *d1*, *d2* present, minute. Length of lobar shield excluding lamellae 81 (76–81). Opisthosomal lobes separated by parallel-sided terminal cleft, length of cleft from anterior end to apices of lamellae 71 (67–73), widest part 16 (16–18). Lamellae with margins indented (7–8 indentations) (Fig. 7). Seta *h2* 233 (230–263) long, seta *h3* 178 (164–197) long.

Coxal apodemes I free. Rudimentary sclerites rEpIIa as thin, oblique bands. Humeral shield ventrally fused with EpIII. Genital apparatus situated between levels of trochanters III, IV, length 37 (33–37), greatest width 12 (10–13) (Fig. 7b). Small, round epiandrum present. Postgenital plaque absent, setae g thin, piliform. Adanal apodemes heavily sclerotized, with small ventral rounded apophyses. Translobar apodeme absent. Adanal shields present, bearing setae ps3. Anal suckers 16 (16–17) in diameter, distance between centers of discs 35 (32–35). Coxal apodemes IVa small, not reaching level of setae *4a*. Setae *4b* situated anterior to level of setae *3a*, setae *g* anterior to *4a*.

Legs IV extending by ambulacral disc to level of setae *h3*. Setae *sR* of trochanters III short, narrowly lanceolate, acute apically, 31 (28–31) long. Modified setae *d* of tarsus IV barrel-shaped, with discoid cap, situated apically; modified setae *e* hemispheroid, without cap, situated apically.

***Female.*** [Range for 5 paratypes.] Length of idiosoma from anterior end to apices of lamellar lobar processes 423–442, greatest width 239–246. Length of hysterosoma from sejugal furrow to apices of lamellar lobar processes 268–282. Prodorsal shield: shaped as in male, 131–137 in length, 179–189 in width, surface as in the male (Fig. 8a). Vertical setae *ve* represented only by alveoli. Setae *si* thin piliform, 20–23 long, separated by 79–85; setae *se* 139–161 long, separated by 126–130. Humeral shields with setae *c2* thin, 32–42 long. Setae *c3* narrowly lanceolate, acute apically, 22–24 in length. Hysteronotal shield: length from anterior margin to bases of setae *h3* 262–269, width at largest part near anterior margin 174–186, lateral margins with incisions at level of trochanters III, DHA present, with small elongate lacunae from level of setae *d1* to *d2*, and two main longitudinal rows of 3–4 large elongate lacunae between the level of setae *e1* to *e2* (Fig. 8a). Dorsal setae *d1*, *d2* present. Setae *h1* thin piliform, 7–10 long, situated antero-mesal to bases of setae *h2*. Width of opisthosoma at level of setae *h2* 80–82. Setae *ps1* positioned dorsally on opisthosomal lobes, closer to bases of *h3* than to *h2*, equidistant from outer and inner margins of lobe. Distance from bases of setae *h3* to membranous apices of lobes 16–18. Setae *f2* indistinct. Supranal concavity open posteriorly into terminal cleft. Length of terminal cleft together with supranal concavity 65–68, width of cleft at level of setae *h3* 11–21. Interlobar membrane thin. External copulatory tube absent. Setae *h2* 234–277 long, setae *h3* 176–206 long.

Coxal apodemes I free. Epigynum 36–39 in length, 95–104 in width (Fig. 8b). Coxal apodemes IVa absent. Setae *sR* of trochanters III narrowly lanceolate, acute apically, 29–34 long. Legs IV extending by ambulacral disc to level slightly posterior to lobes.

### Differential diagnosis

*Trouessartia transatlantica* n. sp. belongs to the *estrildae* species group [20] and is very close to *T. decorata* Gaud & Mouchet, 1958, in having, in males, well-separated lobes, postgenital plaque absent, and translobar apodeme absent. It can be distinguished from the latter species by the following features: in males, hysterosomal shield smooth; in females, small elongate lacunae between setae *d1* and *d2*, and two distinct rows of 3–4 elongate lacunae between setae *e1* and *e2*. In *T. decorata*, males have numerous circular lacunae on the hysteronotal shield, and females have large circular lacunae between *d1* and *d2* and two distinct rows of 7–8 elongate lacunae between *e1* and *e2*.

***Trouessartia minuscula*** **Gaud & Mouchet, 1958**

***Host*** **:** *Estrilda nonnula* Hartlaub, 1883, *E. melpoda* (Vieillot, 1817), *E. astrild* (Passeriformes: Estrildidae).

***Localities*** **:** Brazil, Cameroon, D.R. Congo, Ethiopia, Gabon, Malawi, Mauritius, Mozambique, South Africa, South Sudan, Uganda.

***Type-material examined*** **:** Holotype male ex *Estrilda nonnula* Hartlaub, 1883 (Passeriformes: Estrildidae), CAMEROON: Yaoundé, November 1950, J. Mouchet col. (MRAC # 185.428); paratypes 3 females ex *Estrilda melpoda* (Vieillot, 1817), same locality and collector, November 1956, (MRAC # 185.429; 185.431). Type-specimens deposited in MRAC.

***Voucher material*** **:** Material examined. 3 males and 8 females ex *Estrilda astrild* (Linnaeus, 1758) (Passeriformes: Estrildidae), BRAZIL: São Paulo State, Campus of UNESP, Rio Claro, 22°24′S, 47°33′W, 6.v.2015, M.H. Gabriel coll.; 2 females, same host species, BRAZIL: Rio Claro, São Paulo State, 13 January 2016, C.O.A. Gussoni coll.; one female ex *E. astrild*, SOUTH AFRICA: Limpopo, Haenertsburg, 26–27.xi.1961 (MRAC # 185.430); 1 male ex *E. a. rubriventris* (Vieillot, 1817), GABON: Ngounié, Agouma, River Nkomi, 01°32′29″S, 10°11′14″E, 7.xii.1917, C.R. Aschemeier, USNM 255788 (UGA 3138); 2 females ex *E. a. rubriventris*, same data as previous, 24.xi.1918, USNM 255787 (UGA 3139); 7 males and 1 female ex *E. a. peasei* Shelley, 1903, ETHIOPIA: Oromiya, Aleta, 09°46′N, 38°45′E, 11.iii.1912, E.A. Mearns, USNM 247464 (UGA 3142); 1 female ex *E. a. peasei*, same data as previous, USNM 247463 (UGA 3143); 1 male ex *E. a. macmillani* Ogilvie-Grant, 1907, SOUTH SUDAN: Jubek State, Gondokoro, 04°54′09″N, 31°39′46″E, 23.ii.1910, E.A. Mearns, USNM 217329 (UGA 3145); 3 males ex *E. a. macmillani*, same data as previous, 21.ii.1910, USNM 217327 (UGA 3146); 1 male ex *E. a. astrild* (Linnaeus, 1758), SOUTH AFRICA: Western Cape Prov., Mossel Bay. 34°10′59″S, 22°07′42″E, 8.xii.1953, F. Zumpt (NU 3595); 1 female ex *E. a. cavendishi* Sharpe, 1900, MOZAMBIQUE: Sofala Prov., Búzi, NW Beira, 1952′53″S, 34°36′03″E, 8.xi.1961 (NU 4190); 1 male and 1 female ex *E. a. cavendishi*, MALAWI: Northern Region, Rumphi dist., Khuta maji, Vwaza Marsh, Vwaza Wildlife Reserve, 1170 m, 10°52′S, 33°27′E, 1.x.2009, J.M. Bates, FMNH 489300 (BMOC 10–0503-001); 3 males ex *E. a. cavendishi*, same data as previous, 14.x.2009, J.W. Weckstein, FMNH 489301 (BMOC 10–0503-003); 2 males and 2 females ex *E. a. cavendishi*, MAURITIUS: Rodrigues Is. 19°42′S, 63°25′E, 1.x.1964, F.B. Gill, USNM 486951 (UGA 3173, 3174); 1 male and 2 females ex *E. a. cavendishi*, French Overseas Department: Reunion Island, Étang-Salé les Bains, 21°16′05″S, 55°20′03″E, 19.ix.1964, F.B. Gill, USNM 487040 (UGA 3176). Voucher specimens from *E. astrild* of Brazil deposited in DZUnesp-RC (# 3835–3847). African specimens in FMNH, UMMZ, USNM.

### Remarks

**Fig. 9:**
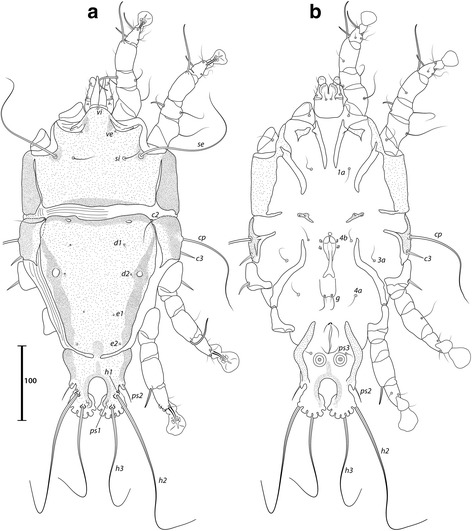
*Trouessartia minuscula* Gaud & Mouchet, 1958, male. **a** Dorsal view. **b** Ventral view

**Fig. 10:**
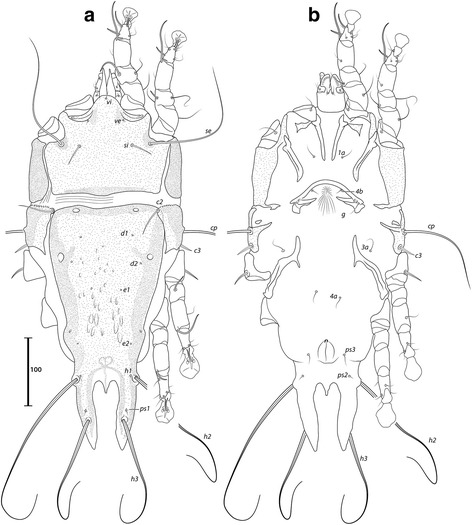
*Trouessartia minuscula* Gaud & Mouchet, 1958, female. **a** Dorsal view. **b** Ventral view

Described from *Estrilda nonnula* Hartlaub (Estrildidae) from Cameroon [29], this species is readily recognized by the conspicuous dark edges of the dorsal shields in both sexes, and by the bifurcate seta *g* of males (Figs. 9 and 10). Santana [20] expressed doubts concerning the association between the only male from the type-host and the few females from *E. melpoda* (Vieillot, 1817). Here, in addition to the aforementioned material deposited at the MRAC, we analysed specimens from *E. astrild* from South Africa and Brazil, with males and females from this latter locality, and can confirm the association between both sexes of this species. The female of *T. minuscula* is herein illustrated for the first time, and *Estrilda astrild* represents a new host for this mite species.

***Trouessartia estrildae*** **Gaud & Mouchet, 1958**

***Host*** **:** *Estrilda nonnula*, *E. astrild*.

***Localities*** **:** Cameroon, Brazil, D.R. Congo, Ethiopia, Kenya, Malawi, South Sudan, South Africa, Uganda.

***Type-material examined*** **:** Holotype female ex *Estrilda nonnula* Hartlaub, 1883 (Estrildidae), Cameroon: Yaoundé, November 1950, J. Mouchet col. (MRAC # 185.292). Type-specimens deposited in MRAC.

***Voucher material*** **:** 1 male and 1 female ex *Estrilda astrild* (Linnaeus, 1758) (Passeriformes: Estrildidae), BRAZIL: São Paulo State, Campus of UNESP, Rio Claro, 22°24′S, 47°33′W, 6.v.2015, M.H. Gabriel coll.; 2 males and 4 females, same host species, BRAZIL: Rio Claro, São Paulo State, 13.i.2016, C.O.A. Gussoni coll.; 2 males and 3 females ex *E. a. adesma* Reichenow, 1916, D. R. CONGO: South Kivu, Centre de Recherche en Sciences Naturelles, Lwiro, 1702 m, 02°14′S, 28°48′E, 13.vi.2001, J.S. Hunt, (JSH 031), FMNH 429841 (BMOC 02-0625-011); 1 female ex *E. a. peasei* Shelley, 1903, ETHIOPIA: Oromiya, Aleta, 09°46′N, 38°45′E, 11.iii.1912, E.A. Mearns, USNM 247464 (UGA 3142); 1 male ex *E. a. macmillani* Ogilvie-Grant, 1907, SOUTH SUDAN: Jubek State, Gondokoro, 04°54′09″N, 31°39′46″E, 23.ii.1910, E.A. Mearns, USNM 217328 (UGA 3144); 1 female ex *E. a. macmillani*, same data as previous, USNM 217329 (UGA 3145); 2 males ex *E. a. macmillani*, same data as previoius, 21.ii.1910, USNM 217327 (UGA 3146); 1 male ex *E. a. astrild* (Linnaeus, 1758), SOUTH AFRICA: Western Cape Prov., Mossel Bay, 34°10′59″S, 22°07′42″E, 8.xii.1953, F. Zumpt, (NU 3604); 2 males and 3 females ex *E. a. cavendishi* Sharpe, 1900, MOZAMBIQUE: Sofala Prov., Búzi, NW Beira, 19°52′53″S, 34°36′03″E, 8.xi.1961 (NU 4190); 1 male and 1 female ex *E. a. cavendishi*, MALAWI: Northern Region, Rumphi dist., Khuta maji, Vwaza Marsh, Vwaza Wildlife Reserve, 1170 m, 10°52′S, 33°27′E, 1.x.2009, J.M. Bates, FMNH 489300 (BMOC 10–0503-001); 1 male and 1 female ex *E. a. cavendishi*, same data as previous, 14.x.2009, J.W. Weckstein, FMNH 489301 (BMOC 10–0503-003). Voucher specimens from *E. astrild* of Brazil deposited in DZUnesp-RC (# 3848–3855). African specimens in FMNH, UMMZ, USNM.

### Remarks

**Fig. 11:**
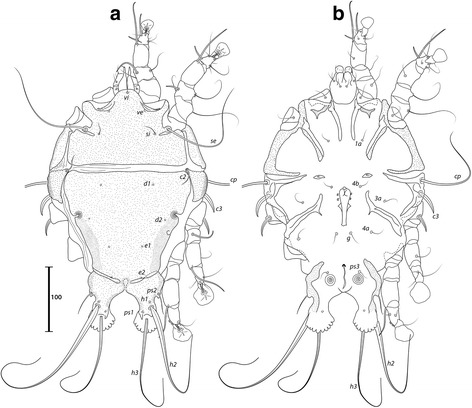
*Trouessartia estrildae* Gaud & Mouchet, 1958, male. **a** Dorsal view. **b** Ventral view

**Fig. 12:**
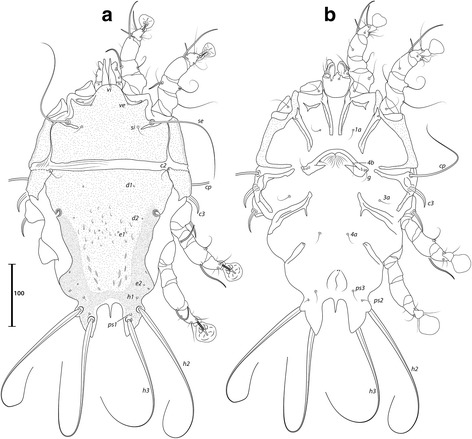
*Trouessartia estrildae* Gaud & Mouchet, 1958, female. **a** Dorsal view. **b** Ventral view

Described from *Estrilda nonnula*, this species is reported on *E. astrild* for the first time. It is readily recognized in both sexes by the enlarged, curved setae *c3* and *sR*-III, and by the incision on the prodorsal shield immediately posterior to setae *se* (Figs. 11 and 12).

**Family Pyroglyphidae Cunliffe, 1958**

**Subfamily Onychalginae Fain, 1988**

**Genus** ***Onychalges*** **Gaud & Mouchet, 1959**

***Onychalges pachyspathus*** **Gaud, 1968**

***Hosts*** **:** *Estrilda melpoda*, *E. atricapilla*, *E. nonnula*, *E. astrild*.

***Localities*** **:** Brazil, Gabon, Mozambique, Malawi.

***Type-material examined*** **:** Holotype male, 2 male and 2 female paratypes ex *Estrilda melpoda* (Vieillot, 1817) (Passeriformes: Estrildidae), CAMEROON, xi.1955, J. Mouchet coll. (MRAC 188.256), deposited in MRAC.

***Voucher material*** **:** 2 males, 15 females and 4 nymphs ex *Estrilda astrild* (Linnaeus, 1758) (Passeriformes: Estrildidae), BRAZIL: Campus of UNESP, Rio Claro, São Paulo State, 22°24′S, 47°33′W, 6.v.2015, M.H. Gabriel coll.; 1 male ex *E. a. rubriventris* (Vieillot, 1817), GABON: Ngounié, Agouma, River Nkomi, 01°32′29″S, 10°11′14″E, 7.xii.1917, C.R. Aschemeier, USNM 255788 (UGA 3138); 1 male and 12 females ex *E. a. adesma* Reichenow, 1916, D. R. CONGO: South Kivu, Centre de Recherche en Sciences Naturelles, Lwiro, 1702 m, 02°14′S, 28°48′E, 13.vi.2001, J.S. Hunt (JSH 031), FMNH 429841 (BMOC 02–0625-011); 1 male and 1 female ex *E. a. cavendishi* Sharpe, 1900, MOZAMBIQUE: Sofala Prov., Búzi, NW Beira, 19°52′53″S, 34°36′03″E, 8.xi.1961 (NU 4190); 2 males and 1 female ex *E. a. cavendishi*, MALAWI: Northern Region, Rumphi dist., Khuta maji, Vwaza Marsh, Vwaza Wildlife Reserve, 1170 m, 10°52′S, 33°27′E, 1.x.2009, J.M. Bates, FMNH 489300 (BMOC 10–0503-001); 2 males ex *E. a. cavendishi*, same data as previous, 14.x.2009, J.W. Weckstein, FMNH 489301 (BMOC 10–0503-003). Voucher specimens from *E. astrild* of Brazil deposited in DZUnesp-RC (# 3856–3872) and UMMZ. African specimens in FMNH, UMMZ, USNM.

### Remarks

**Fig. 13:**
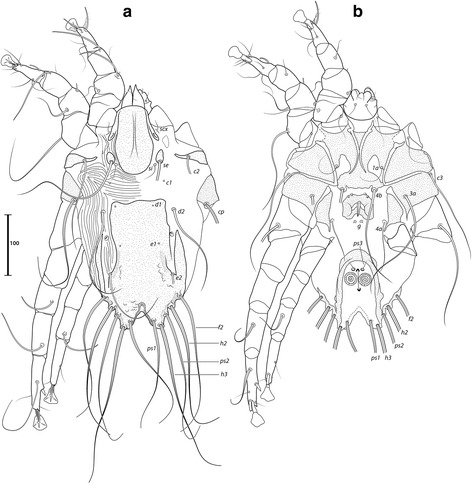
*Onychalges pachyspathus* Gaud, 1968, male. **a** Dorsal view. **b** Ventral view

**Fig. 14:**
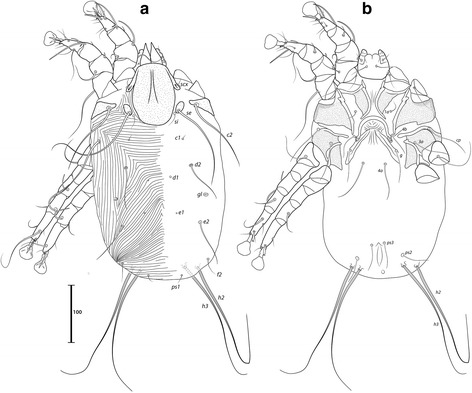
*Onychalges pachyspathus* Gaud, 1968, female. **a** Dorsal view. **b** Ventral view

This species has been reported from passerines of the genus *Estrilda* (Estrildidae): *E. melpoda* (Vieillot) (type-host), *E. nonnula* Hartlaub, *E. atricapilla* Verreaux & Verreaux, and *E. astrild* (Linnaeus) [4, 31] (Figs. 13 and 14).

**Family Dermationidae Fain, 1965**

**Subfamily Dermationinae Fain, 1965**

**Genus** ***Paddacoptes*** **Fain, 1964**

***Paddacoptes paddae*** **(Fain, 1964)**

***Hosts*** **:** *Lonchura oryzivora* (Linnaeus, 1758) (type-host), *L. punctulata* (Linnaeus, 1758), *L. malacca* (Linnaeus, 1766), *L. cucullata* (Swainson, 1837), *Estrilda astrild*.

***Localities*** **:** Indonesia, Java, D.R. Congo, Brazil.

***Voucher material*** **:** 1 male, 28 females ex *Estrilda astrild* (Linnaeus, 1758) (Passeriformes: Estrildidae), BRAZIL: São Paulo State, Campus of UNESP, Rio Claro, 22°24′S, 47°33′W, 6.v.2015, M.H. Gabriel coll; specimens deposited in DZUnesp-RC (# 3873–3896) and UMMZ.

### Remarks

**Fig. 15:**
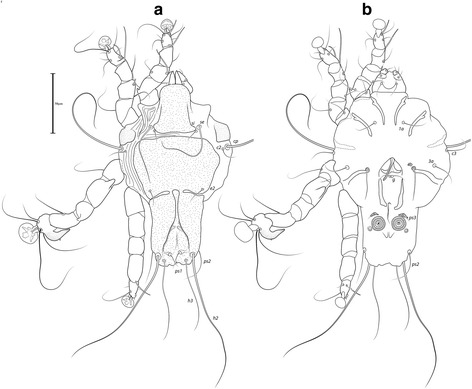
*Paddacoptes paddae* (Fain, 1964), male. **a** Dorsal view. **b** Ventral view

**Fig. 16:**
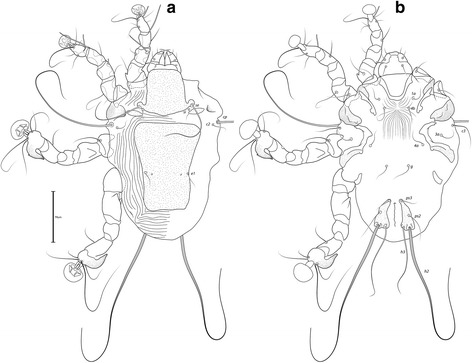
*Paddacoptes paddae* (Fain, 1964), female. **a** Dorsal view. **b** Ventral view

Seven *Paddacoptes* species are known from passerines (Estrildidae, Viduidae) and pigeons (Columbidae) [33]. *Paddacoptes paddae* (Fain, 1964) (Figs. 15 and 16) was described from African and Asian passerines of the family Estrildidae: *Lonchura oryzivora* (Linnaeus, 1758) (type-host), *L. punctulata* (Linnaeus, 1758), *L. malacca* (Linnaeus, 1766), *Spermestes cucullatus* (Swainson, 1837), and *Estrilda atricapilla* (Verreaux & Verreaux, 1851) [33]. It is reported here for the first time on *Estrilda astrild*.

**Order Trombidiformes Reuter, 1909**

**Superfamily Cheyletoidea Leach, 1815**

**Family Cheyletidae Leach, 1815**

**Genus** ***Neocheyletiella*** **Baker 1949**

***Neocheyletiella megaphallos*** **(Lawrence, 1959)**

***Hosts*** **:** *Estrilda erythronotos* (Vieillot, 1817), *E. astrild*.

***Localities*** **:** Botswana, Brazil.

***Voucher material*** **:** 5 males, 9 females and 4 nymphs ex *Estrilda astrild* (Linnaeus, 1758) (Passeriformes: Estrildidae), BRAZIL: São Paulo State,Campus of UNESP, Rio Claro, 22°24′S, 47°33′W, 6.v.2015, M.H. Gabriel coll; specimens deposited in DZUnesp-RC (# 3897–3907) and UMMZ.

### Remarks

**Fig. 17:**
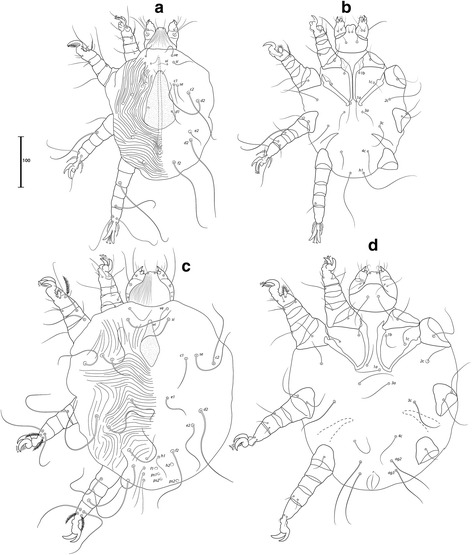
*Neocheyletiella megaphallos* (Lawrence, 1959). **a** Male, dorsal view. **b** Male, ventral view. **c** Female, dorsal view. **d** Female, ventral view

Described from the Black-faced waxbill, *Estrilda erythronotos* (Vieillot, 1817) (Estrildidae), this species bears the longest aedeagus among the 17 species of the genus [32], being longer than half the length of the male’s idiosoma (Fig. 17a). It is recorded on *E. astrild* for the first time.

## Discussion

Prior to this study, the only mite previously known from the common waxbill was *Onychalges pachyspathus* Gaud, 1968 [4]. Herein we add eight more mite species to the list of associates of this bird. The acarofauna found on *Estrilda astrild* from Brazil includes only typical Old World taxa, most of which are thus recorded for the first time in the Neotropical region. Of the nearly 60 known species of *Montesauria*, none is known from the Neotropics, and only one is known from North America [10], while all the remaing species are endemic to either Africa, Eurasia or Australasia. Despite *Trouessartia* species having been found worldwide, mostly on passerines, the three species found on *E. astrild* belong to the *estrildae* group, a group of 12 species restricted to African estrildids [20]. Of the six species of *Onychalges* (Pyroglyphidae), all but one are confined to African estrildids, the exception being *O. nidicola* Fain & Rosa, 1982 described from a nest of *Passer domesticus* (Linnaeus, 1758) (Passeridae) in Brazil [34]. This host, however, is believed to be accidental, and this mite may have entered South America via introduced estrildids [4]. Only two of the seven species of *Paddacoptes* are described from New World pigeons (Columbidae), the remaining species are from African or Australasian passerines of the families Estrildidae and Viduidae [32]. As for the genus *Neocheyletiella*, four species occur on the Estrildidae, and *N. megaphallos* is the only one associated with the genus *Estrilda* [35–38]. Finally, despite no *Xolalgoides* (Xolalgidae) specimens were recovered from the Brazilian specimens, this might simply be due to the low number of waxbill specimens (2) analyzed from this country.

These mites represent at least three morpho-ecological groups regarding their microhabitats occupied on the bird: (i) vane mites, *Montesauria* and *Trouessartia*, on the ventral and dorsal surfaces of the large wing feathers, respectively, and also on the tail feathers; (ii) down mites (*Onychalges*); and (iii) skin mites (*Paddacoptes*, *Neocheyletiella*). The finding of eight mite species on a single host, including two and three congeners (*Montesauria* and *Trouessartia*, respectively) gives a perspective on how diverse the acariform fauna associated with passerines is. On one specimen of the Common waxbill in Brazil we found representatives of all eight mite species.

*Estrilda astrild* is believed to have been brought from Africa to Brazil in slave ships between 1822 and 1831, during the Atlantic slave trade [1]. It normally feeds on introduced African grasses and build their own nests (i.e. do not use cavity nests built by other birds), and thus offers limited competition to, or at least little interaction with native birds of Brazil [1]. This offers few opportunities for horizontal transfer of mites. Indeed, after almost two centuries in the new environment, the common waxbill has probably not yet acquired any representatives of typical Neotropical mite taxa.

Feather mites usually tend to remain associated only with their native or closely related hosts even when are introduced to other regions [17]. Thus, commonly introduced birds in Brazil, either reared as pets e.g. cockatiels, *Nymphicus hollandicus* (Kerr, 1792), budgerigars, *Melopsittacus undulatus* (Shaw, 1805) or poultry [e.g. domestic chickens, *Gallus gallus domesticus* Linnaeus, 1758], or those now living in the wild, e.g. house sparrows, *Passer domesticus* Linnaeus, 1758 and rock pigeons, *Columba livia* Gmelin, 1789, still have the same mites as reported from their native range. In these cases *Nymphicilichus perezae* Mironov & Galloway, 2002 (Pterolichidae) persists on cockatiels, *Sideroferus lunula* (Robin, 1877) (Pterolichidae) on budgerigars, *Megninia cubitalis* (Mégnin, 1877) and *M. ginglymura* (Mégnin, 1877) (Analgidae) on farmed chickens, *Proctophyllodes troncatus* Robin, 1877 (Proctophyllodidae) on house sparrows, and *Falculifer rostratus* (Buchholz, 1869) (Falculiferidae) on rock pigeons ([39–41], FAH, pers. obs.).

The recent discovery of horizontal transfer of *Allopsoroptoides galli* from wild Guira cuckoos, *Guira guira* (Gmelin, 1788), Cuculiformes, to laying hens, *Gallus gallus domesticus* (Linnaeus, 1758) (Galliformes), in Brazil demonstrates that feather mites may occasionally colonize phylogenetically distant hosts, especially in captivity [42]. In the case of the mites from *Estrilda astrild*, we suspect that this would be a highly improbable event, given the fact that most feather mites are highly host specific, and the common waxbill remains the only representative of the family Estrildidae introduced in the Neotropics.

## Conclusion

In this study, eight mites belonging to five families and two orders are reported for the first time from *Estrilda astrild* from its introduced range in Brazil, all of which are recorded for the first time in the Neotropics. We conclude that the mite community reported from this bird species contains only typical Old World taxa, despite this bird having been present in the new environment for almost two centuries. No representatives of Neotropical acarine taxa were found associated with this bird. We suspect that the horizontal transfer between these distinct acarofaunas would be quite an unlikely event, since most feather mites are highly host specific, and the common waxbill remains the only estrildid introduced in the Neotropics.
